# Combined antibiotic stewardship and infection control measures to contain the spread of linezolid-resistant *Staphylococcus epidermidis* in an intensive care unit

**DOI:** 10.1186/s13756-021-00970-3

**Published:** 2021-06-30

**Authors:** Cihan Papan, Matthias Schröder, Mathias Hoffmann, Heike Knoll, Katharina Last, Frederic Albrecht, Jürgen Geisel, Tobias Fink, Barbara C. Gärtner, Alexander Mellmann, Thomas Volk, Fabian K. Berger, Sören L. Becker

**Affiliations:** 1 Center for Infectious Diseases, Institute of Medical Microbiology and Hygiene, Saarland University, Kirrberger Strasse, Building 43, 66421 Homburg, Germany; 2 Department of Anesthesiology, Intensive Care and Pain Therapy, Saarland University Medical Center, Homburg, Germany; 3 Hospital Pharmacy, Saarland University, Homburg, Germany; 4 Department of Clinical Chemistry and Laboratory Medicine, Saarland University, Homburg, Germany; 5 Institute of Hygiene, University Hospital Münster, Münster, Germany

**Keywords:** Antimicrobial stewardship, Infection control, Antimicrobial resistance, Linezolid, Staphylococci, Whole-genome sequencing

## Abstract

**Background:**

The unrestricted use of linezolid has been linked to the emergence of linezolid-resistant *Staphylococcus epidermidis* (LRSE). We report the effects of combined antibiotic stewardship and infection control measures on the spread of LRSE in an intensive care unit (ICU).

**Methods:**

Microbiological data were reviewed to identify all LRSE detected in clinical samples at an ICU in southwest Germany. Quantitative data on the use of antibiotics with Gram-positive coverage were obtained in defined daily doses (DDD) per 100 patient-days (PD). In addition to infection control measures, an antibiotic stewardship intervention was started in May 2019, focusing on linezolid restriction and promoting vancomycin, wherever needed. We compared data from the pre-intervention period (May 2018–April 2019) to the post-intervention period (May 2019–April 2020). Whole-genome sequencing (WGS) was performed to determine the genetic relatedness of LRSE isolates.

**Results:**

In the pre-intervention period, LRSE were isolated from 31 patients (17 in blood cultures). The average consumption of linezolid and daptomycin decreased from 7.5 DDD/100 PD and 12.3 DDD/100 PD per month in the pre-intervention period to 2.5 DDD/100 PD and 5.7 DDD/100 PD per month in the post-intervention period (p = 0.0022 and 0.0205), respectively. Conversely, vancomycin consumption increased from 0.2 DDD/100 PD per month to 4.7 DDD/100 PD per month (p < 0.0001). In the post-intervention period, LRSE were detected in 6 patients (4 in blood cultures) (p = 0.0065). WGS revealed the predominance of one single clone.

**Conclusions:**

Complementing infection control measures by targeted antibiotic stewardship interventions was beneficial in containing the spread of LRSE in an ICU.

**Supplementary Information:**

The online version contains supplementary material available at 10.1186/s13756-021-00970-3.

## Background

*Staphylococcus epidermidis* belongs to the group of coagulase-negative staphylococci and is a major constituent of the human skin flora [1]. Its detection in microbiological samples is often interpreted as contamination and/or colonization. In nosocomial infections, however, *S*. *epidermidis* can play an important role, especially in immunocompromised patients or those with indwelling catheters or other foreign bodies [2, 3]. Treatment of clinically relevant *S*. *epidermidis* infections frequently requires the use of glycopeptides, oxazolidinones or lipopeptide antibiotics, as resistance to beta-lactams is widespread among *S*. *epidermidis* strains [4].

The oxazolidinone antibiotic linezolid acts as an inhibitor of bacterial protein synthesis, and was approved for the treatment of infections caused by Gram-positive organisms in 2000 [5]. While vancomycin has remained the drug of choice for infections caused by methicillin-resistant *Staphylococcus aureus* (MRSA), linezolid has gained importance as an alternative, especially in MRSA pneumonia, but also in infections caused by vancomycin-resistant enterococci (VRE) [6]. There is no associated nephrotoxicity related to the use of linezolid, making it a favoured antibiotic especially in critically ill patients with impaired renal function. In contrast, the nephrotoxicity encountered with the use of vancomycin requires stringent therapeutic drug monitoring, to ensure efficient drug levels while at the same time avoiding toxicities.

While the first reports on linezolid resistance were attributed to spontaneous point mutations [7], which mainly affect the 23S rRNA binding site or the 50S ribosomal proteins, there has been a more recent surge in reports on mobile, transferable resistance mechanisms, such as the *cfr* gene [8], or the *optrA* gene [9], the latter being more often attributed to enterococci. Factors postulated to confer risk for linezolid resistance are disease severity, length of intensive care unit (ICU) stay, and exposure to linezolid [10–13]. According to one systematic review with meta-analysis from 2020, the global prevalence of linezolid resistance is reportedly still rather low [14]. Several outbreaks and clusters however, have indicated that especially linezolid-resistant *S. epidermidis* (LRSE) can manifest as an endemic pathogen in ICUs and that these are linked to a preceding linezolid overuse in most cases [10, 15–18].

Here, we report the spread of LRSE in an ICU in southwest Germany, including an in-depth genetic characterization of isolates, and describe the effects of infection control and antimicrobial stewardship measures.

## Methods

### Design, study population, and setting

This report was designed as a before-after study to investigate the effects of infection control and antimicrobial stewardship interventions [19]. The reporting adhered to the ORION guidelines [20]. We compared the pre-intervention period from May 2018 to April 2019 with the post-intervention period from May 2019 to April 2020.

A positive case was defined as having a LRSE in any clinical specimen, excluding screening specimens. Each patient was included only once, and only a single isolate was investigated per patient. Linezolid resistance was diagnosed when the confirmatory testing yielded a minimal inhibitory concentration (MIC) of > 4 mg/L, in accordance with the guidelines of the European Committee on Antimicrobial Susceptibility Testing (EUCAST). We performed a subgroup analysis on patients with LRSE detected in blood cultures. The intervention was performed on a predominantly surgical intensive care unit with 26 beds at the Saarland University Medical Center, a large university hospital in southwest Germany, with a total of 1288 beds.

### Interventions

Infection control practices were implemented in July 2018, including isolation and cohorting of patients, as well as contact precaution comprising glove and gown use in the care of patients with LRSE. At the same time, a catheter-care bundle was initiated, comprising the development of a standard operating procedure, the use of chlorhexidine-impregnated dressings and specific luer access valve caps with 70% isopropyl alcohol for central venous catheters, and reinforcing a policy to discourage blood cultures from indwelling arterial catheters.

After a review of data on antibiotic use, we conceived an antibiotic stewardship intervention, initiated at the beginning of May 2019, aiming at the reduction of linezolid use and endorsing vancomycin. The intervention consisted of regular audits with feedback, educational meetings, and provision of pocket cards on the use of antibiotics with Gram-positive coverage (Additional file 1: Figure S1, available as Supplementary data). When indicated, vancomycin was recommended, while the use of linezolid, but also daptomycin were discouraged.

### Microbiological diagnostics

All LRSE isolates were detected using standard microbiological procedures. In brief, bacteria were identified after growth on blood agar or other agar media by matrix-assisted laser desorption/ionization time-of-flight mass spectrometry (MALDI-TOF MS) using a Microflex LT mass spectrometer (Bruker Daltonics; Bremen, Germany).

Antimicrobial susceptibility testing was performed employing VITEK II (BioMérieux; Marcy l’Étoile, France) and confirmatory testing with MIC Test Strip (Liofilchem; Roseto degli Abruzzi, Italy). For interpretation, the guidelines of the European Committee on Antimicrobial Susceptibility Testing (EUCAST; versions 9.0 and 10.0) were followed.

### Whole-genome sequencing (WGS) and WGS-based genotyping

Whole-genome sequencing (WGS) of available LRSE was performed using long-read Pacific Biosciences technology (Pacific Biosciences Inc., Menlo Park, CA, USA). In brief, high molecular weight DNA was extracted using Monarch Genomic DNA Purification Kit (New England Biolabs, MA, USA) prior to library preparation using the SMRTbell Express Template Prep Kit 2.0 (Pacific Biosciences Inc.). The DNA polymerase/template complex was prepared using the Sequel Binding Kit 2.1 (Pacific Biosciences Inc.) and sequenced on a Sequel II system (Pacific Biosciences Inc.). The resulting sequences were assembled using the microbial assembly pipeline integrated in the SMRT Link version 8 software with default parameters (Pacific Biosciences Inc.). Gene sequences were extracted for subsequent core genome multilocus sequence typing (cgMLST)-based typing using an ad hoc cgMLST scheme consisting of 1,846 genes (Additional file 2: Table S1, available as Supplementary data) and SeqSphere^+^ software version 6 (Ridom GmbH, Münster, Germany) using default parameters as described elsewhere [21]. For backwards compatibility with previous typing efforts, the MLST sequence types (STs) were also extracted with the help of the SeqSphere^+^ software.

To determine the genetic basis of the linezolid resistance, we analysed the genome sequences using the NCBI AMRFinderPlus [22] integrated in the SeqSphere^+^ software.

### Outcomes

Detection of LRSE in blood cultures and other clinical specimens was analysed on a monthly basis. In addition, we calculated medians per month to account for variability. We investigated the quarterly trends (i.e. for three consecutive months each) of LRSE rates, both, per *S*. *epidermidis* in all specimens (LRSE per *S*. *epidermidis*), and per patient activity (per 10,000 patient-days) for both time periods. May to July 2018 was designated as quarter (Q) 1.1, August to October 2018 as Q1.2, November 2018 to January 2019 as Q1.3, and February 2019 to April 2019 as Q1.4. Likewise, the quarters for the post-intervention period were designated as Q2.1, 2.2, 2.3, and 2.4. Antibiotic consumption was assessed by calculating defined daily doses (DDD), according to the ATC/DDD index of the WHO Collaborating Centre for Drug Statistics Methodology, per 100 patient-days (PD) per month for intravenous vancomycin (DDD = 2 g), intravenous linezolid (DDD = 1.2 g), and intravenous daptomycin (DDD = 0.28 g). For the assessment of time trends, we calculated slope changes. To assess whether the propagated use of vancomycin was associated with target attainment, therapeutic drug monitoring including the number of vancomycin trough level orders and the respective trough levels were analysed.

We considered potential confounders such as length of stay, case mix, and in-house mortality. In addition, to minimize the risk that a change in ordering behaviour or contamination rates may have influenced the results, the number of ordered blood cultures, and the rate of blood cultures positive with *S. epidermidis* were investigated as well.

### Vancomycin trough levels

Vancomycin trough levels were measured with an immunoassay utilizing a competitive assay format in which microparticles agglutinate (Cobas 8000 c702, Roche Diagnostics; Mannheim, Germany).

### Ethics

Due to the nature of nosocomial infections, all diagnostic and interventional aspects of this work were performed in accordance with the German Protection against Infection Act (Infektionsschutzgesetz). Hence, an ethics approval was not necessary. All patient-related data were anonymized.

### Statistical methods

We performed statistical analyses by using GraphPad Prism (GraphPad Software Inc.; California, USA). We compared the pre-intervention period to the post-intervention period using Mann–Whitney U test and t test for continuous variables; chi-square and Fisher’s exact test for categorical data; and nonlinear regression analysis for discrete changes of antibiotic utilization. The level for statistical significance was set at 0.05.

## Results

### Microbiology

**Fig. 1:**
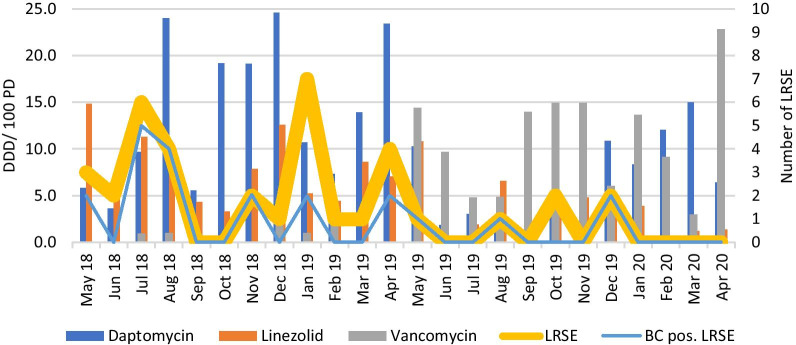
Antibiotic consumption and the incidence of LRSE over time for each month; antibiotic consumptions per substance are shown in columns, the number of LRSE total (yellow) and in blood cultures (blue) as curves. DDD/100 PD: defined daily dose per 100 patient-days; *LRSE* linezolid-resistant *Staphylococcus epidermidis*, *BC* blood culture, *pos.* positive

Between May 1, 2018 and April 30, 2019, 31 patients were identified with LRSE in any clinical specimen (median 2 per month, interquartile range, IQR, 1–4). Of these, 17 had LRSE detected in blood culture (median 1 per month, IQR 0–2). In comparison, in the post-intervention period from May 1st 2019 to April 30th 2020, a total of 6 patients had a detection of LRSE in any specimen (median 0 per month, IQR 0–1) (p = 0.0065), with 4 being positive in blood culture (median 0 per month, IQR 0–0.8) (p = 0.1057) (Table 1) (Fig. 1). Analysis of quarterly trends or LRSE per *S*. *epidermidis* rates yielded 11/53 (20.8%), 4/53 (7.5%), 10/68 (14.7%), and 6/70 (8.6%) cases for Q1.1, Q1.2, Q1.3, and Q1.4, showing a decrease in the post-intervention period, with 1/66 (1.5%), 3/53 (5.7%), 2/53 (3.8%), and 0/60 (0%) cases respectively during Q2.1, Q2.2, Q2.3, and Q2.4. Likewise, LRSE rates per patient activity yielded a decrease, from 57.4/10,000 PD, 22.2/10,000 PD, 57.0/10,000 PD, and 31.6/10,000 PD during the pre-intervention quarters, to 5.2/10,000 PD, 16.1/10,000 PD, 10.6/10,000 PD, and 0/10,000 PD in the post-intervention period quarters, respectively.

**Table 1:** Comparative main study outcomes during the pre- and post-intervention study periods in an investigation of a spread of linezolid-resistant *Staphylococcus epidermidis* on an intensive care unit in southwest Germany, 2018–2020

	Pre-intervention	Post-intervention	p-value
*Microbiological outcomes*			
LRSE in any specimen, median per month (IQR)	2 (1–4)	0 (0–1)	**0.0065**
LRSE in blood culture, median per month (IQR)	1 (0–2)	0 (0–0.8)	0.1057
*Antibiotic consumption outcomes*			
Median linezolid consumption, in DDD/100 PD per month (IQR)	7.5 (4.6–10.8)	2.5 (0.9–4.8)	**0.0022**
Median daptomycin consumption, in DDD/100 PD per month (IQR)	12.3 (6.2–22.4)	5.7 (1.4–10.7)	**0.0205**
Median vancomycin consumption, in DDD/100 PD per month (IQR)	0.2 (0–0.4)	4.7 (2–6)	** < 0.0001**
Combined median antibiotic consumption for all three substances, in DDD/100 PD per month (IQR)	21.9 (13.8–29.6)	14.8 (8.0–17.6)	**0.0233**
*Therapeutic drug monitoring*			
Number of vancomycin trough level measurements	16	221	
Median trough level, in µg/mL (IQR)	10.2 (8.1–13.7)	12.6 (10.2–16.2)	** < 0.0001**
Target attainment (i.e. 10- < 20 µg/mL) (%)	6/16 (37.5%)	144/221 (65.2%)	**0.0331**
*Potential confounders*			
Total number of blood cultures taken	1650	1668	
Blood cultures positive for *Staphylococcus epidermidis*, n (%)	175 (10.6%)	156 (9.4%)	0.2466
Length of stay, days (mean)	4.9	5.6	0.23
Case-mix-index (mean)	4.9	5.3	0.11
In-house mortality in % (no. of deaths per no. of ICU patients)	10.8	10.7	0.95

p-values of significant differences are shown in bold

*LRSE* linezolid-resistant *Staphylococcus epidermidis*, *IQR* interquartile range, *DDD* defined daily doses, *PD* patient-days, *no.* number

Table 2 shows the annual trend before the intervention, as compared to all other departments of the same hospital.

**Table 2:** Percentage of linezolid-resistant strains among all *Staphylococcus epidermidis* in all materials over the time, counted once per patient, for the index ICU in comparison with the rest of the hospital, between 2014 and 2020

	2014 (%)	2015 (%)	2016 (%)	2017 (%)	2018 (%)	2019 (up until April 30th) (%)	2019 (from May 1st) (%)	2020 (up until April 30th) (%)
Index ICU	0	2	1	7	10	8	4	0
Rest of hospital	0	0	0	1	2	3	2	2

### Whole-genome sequencing

**Fig. 2:**
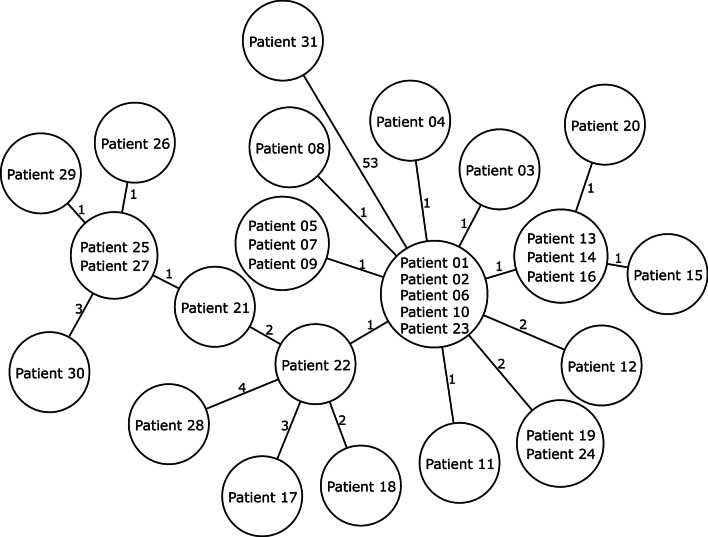
Minimum spanning tree of 31 LRSE isolates. Each circle represents the genotype based on a unique allelic profile of up to 1846 cgMLST genes (ignoring missing values in pairwise comparisons) and the number on connecting lines display the number of differing alleles. The circles are named with the isolates and their size is proportional to the number of isolates with the same genotype

Overall, 31 isolates were subjected to WGS. Whereas all 31 isolates were MLST ST2, WGS revealed that all except one isolate (patient 31) were closely related with ≤ 4 allelic differences in a pairwise comparison to the neighbouring isolate indicating a clonal spread of LRSE. The isolate of Patient 31 is only distantly related to the other 30 LRSE with ≥ 53 alleles differing to all other LRSE isolates of the cluster (Fig. 2). We then screened the genome sequences for the presence of known genes that are associated with linezolid resistance phenotypes, however, we were not able to find any known gene (such as *cfr*) associated with linezolid resistance using the NCBI AMRFinderPlus.

### Antibiotic consumption

Median linezolid consumption during the pre-intervention period was 7.5 DDD/100 patient days (IQR 4.6–10.8), decreasing to 2.5 DDD/100 PD (IQR 0.9–4.8) in the post-intervention period (p = 0.0022) (Table 1) (Fig. 1).

**Fig. 3:**
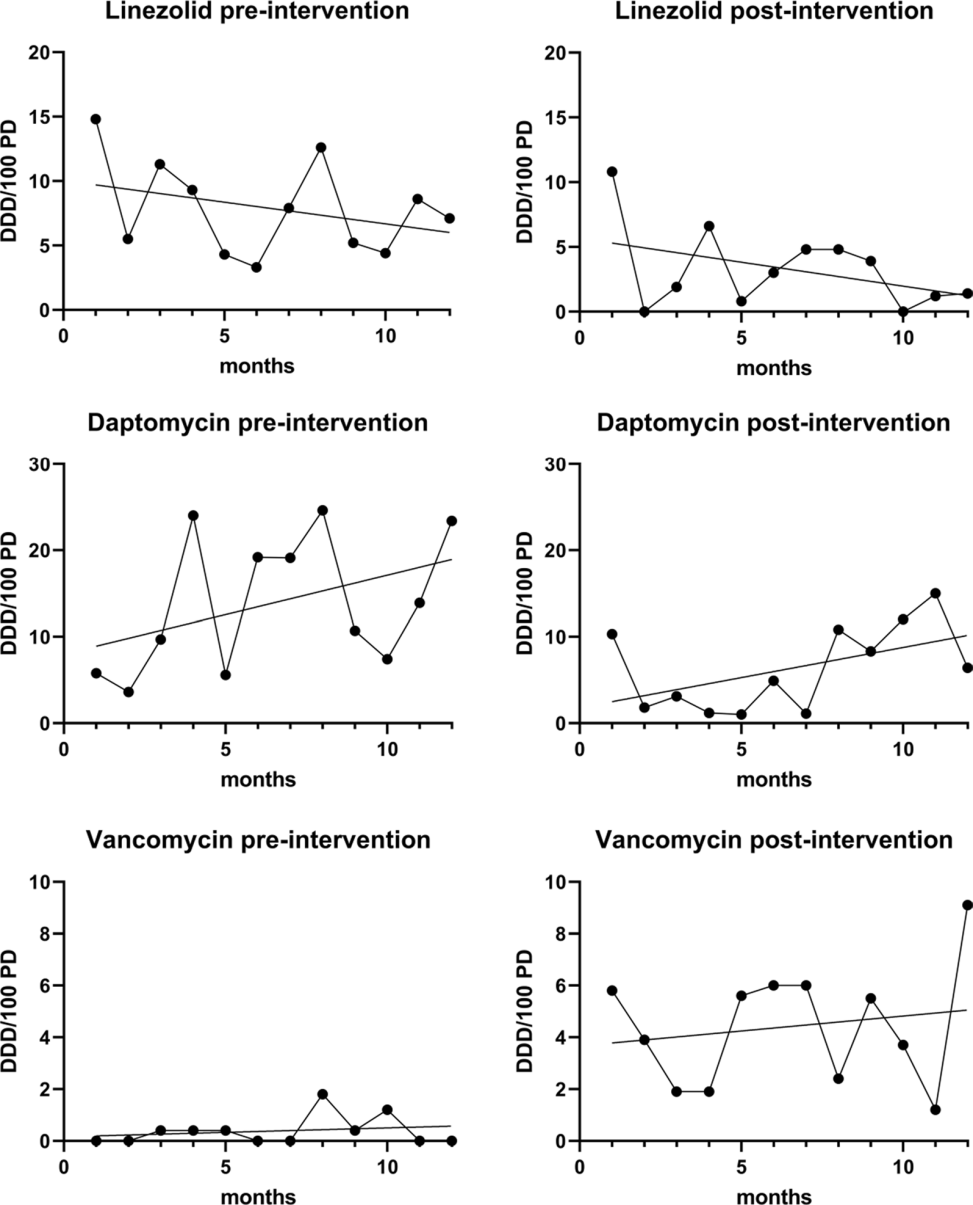
Antibiotic utilization in defined daily dose per 100 patient-days (DDD/100 PD), for each month of both study periods, for linezolid, daptomycin, and vancomycin (from top to bottom)

We observed gradual changes within the pre-intervention period, with a slope of -0.336 (95% confidence interval -1.002 to 0.3296). This downward slope was stronger in the post-intervention period with -0.3692 (95%CI -0.93 to 0.1916) (Fig. 3).

Similarly, daptomycin usage decreased from 12.3 DDD/100 PD (IQR 6.2–22.4) in the pre-intervention period to 5.7 DDD/100 PD (IQR 1.4–10.7) in the post-intervention period (p = 0.0205) (Table 1).

Utilization of vancomycin was promoted, which led to an increase of a median of 0.2 DDD/100 PD (IQR 0–0.4) to 4.7 DDD/100 PD (2–6) (p < 0.0001) (Table 1). Concurrently, the slope increased from 0.03 (95% CI -0.07 – 0.14) in the pre-intervention period to 0.12 (95% CI -0.33 – 0.56) in the post-intervention period.

Detailed analyses of the gradual changes are shown in Fig. 3. Combined utilization of linezolid, daptomycin, and vancomycin decreased significantly (Table 1).

### Vancomycin target attainment

We monitored vancomycin trough level orders and target attainment. Vancomycin trough levels increased from 16 to 221 measurements in the post-intervention period. Median trough levels were 10.2 µg/mL (IQR 8.1–13.7) in the pre-intervention period compared to 12.6 µg/mL (IQR 10.2–16.2) in the post-intervention period (p < 0.0001). Target attainment, defined as a trough level between 10 and < 20 µg/mL, was significantly more often met in the post-intervention period with 144/221 (65.2%) than in the pre-intervention period with 6/16 (37.5%) (p = 0.0331) (Table 1).

### Length of stay, case-mix-indices and mortality

Between the two time periods, we observed no statistically significant difference in mean length of stay, case-mix-indices, mortality, number of blood cultures taken, and the rate of blood cultures positive for all *S. epidermidis* (Table 1). However, the shift in antibiotic utilization led to monetary savings for antibiotics in the magnitude of 16,283.05 € for the year 2019 (Additional file 3: Table S2, available as Supplementary data), disregarding other costs, such as those related to, for example, therapeutic drug monitoring.

## Discussion

Here, we report a spread of LRSE in an ICU linked to and perpetuated by an unrestricted use of linezolid, which we successfully controlled by combined infection control and antimicrobial stewardship measures with sustained effects after one year. Of note, these interventions were not associated with a worse clinical outcome as mortality rates remained unchanged.

Our WGS findings suggest that a single clone was predominant at the ward with 30 of 31 isolates sharing nearly identical genotypes, which in concurrence with the high linezolid use led to an extensive spread, but was at the same time reversible by significantly reducing linezolid utilization rates.

Interestingly, although 30 of 31 isolates clearly belonged to the same single clone, we detected 20 different genotypes with a maximum difference of 10 alleles within the cluster (Fig. 2). This, however, could be explained by the fact that the outbreak period was spanning a longer time period, which very likely enabled LRSE to further diversify. Such micro-evolutionary events frequently take place in prolonged outbreaks such as shown for *Listeria monocytogenes* [23] or *Staphylococcus aureus* [24]. Several nosocomial outbreaks of LRSE have been reported in the past years, most of which focused on the molecular epidemiology and genetic characterization of the microorganisms [10, 15–18]. Descriptions of antimicrobial stewardship interventions are scarce [12] or lack details that would facilitate reproducibility [25–27].

The causal link between linezolid overuse and emergence of linezolid resistance had been demonstrated previously [25, 26]. Mulanovich and colleagues postulated that exceeding a certain threshold of selection pressure, i.e. 13 DDD/100 PD would be necessary for the occurrence of an outbreak [28]. Of note, we observed that the continuity of an outbreak may depend on even lower DDD thresholds, notwithstanding appropriate infection control measures.

While antibiotic cycling, i.e. the projected shift in antibiotics used in a certain unit over time, has been controversially debated with high-quality evidence showing no benefit [29], a change in antibiotic treatment strategy has been demonstrated to be effective in outbreaks with other organisms as well [30]. Here, vancomycin was promoted as first-line antibiotic for patients with presumed infection due to Gram-positive, beta-lactam-resistant bacteria.

The strengths of our study are the ambidirectional design, allowing for a prospective evaluation of the antimicrobial stewardship intervention; the length of the study period to account for secular time trends [31]; and the availability of isolates for WGS analysis to prove genetic linkage.

Some limitations deserve mentioning. We did not perform routine colonization surveillance of LRSE, which would have helped to estimate the effect of the interventions on the colonization prevalence. In the absence of an established and common screening method for linezolid-resistant bacteria, it is conceivable that the actual number of patients with LRSE colonization was higher than our numbers reflected. Furthermore, our report lacks investigations of environmental surfaces and health-care workers (HCW), albeit plausible that the latter could have been a vector in most cases. Still, it appears reasonable to postulate that, even in the absence of a pre-colonization with LRSE, linezolid may have suppressed resident microbiota and thereby enabled transmission and infection by LRSE, as also stated by Weßels and colleagues [25]. Moreover, it can be argued that attributing an outcome to one specific cause is hampered by the multifaceted nature of a combined intervention, comprising infection control measures such as the catheter-care bundle, and antimicrobial stewardship measures. Finally, we assessed DDDs, but not individual lengths or days of therapy, thereby potentially introducing a minor degree of imprecision. Nevertheless, linezolid consumption quantified by DDD for the overall hospital remained stable from mid-2018 through mid-2020 (data not shown), thus refuting a secular trend which could have been underlying the observed changes within the index ward.

The success of our intervention may have been influenced by the nature and depth of the antimicrobial stewardship intervention, i.e. educational efforts put in the endorsement of vancomycin, which had been seldom used previously on the index ICU. This was notably underscored by the vancomycin utilization rate, the increased use of therapeutic drug monitoring and the substantial improvement in target attainment during the post-intervention period.

## Conclusions

In conclusion, our report demonstrates a successful LRSE control composed of infection control measures combined with an antimicrobial stewardship intervention. Future studies should evaluate the benefit of colonization surveillance, and address the question whether HCW screening and decolonization strategies may be of additional help in controlling outbreaks or clusters of LRSE.

## Supplementary Information

**Additional file 1: Figure S1.** Pocket card on the use of antibiotics with Gram-positive coverage, provided during the antimicrobial stewardship intervention on an intensive care unit in southwest Germany, 2018–2020.

**Additional file 2: Table S1.** List of core genome genes used for the ad hoc *S. epidermidis* cgMLST scheme. Locus tags designations were taken from the reference strain ATCC12228 (GenBank acc. no. NC_004461).

**Additional file 3: Table S2.** Expenses for the three antibiotics studied during the investigation of an outbreak by linezolid-resistant *Staphylococcus epidermidis* on an intensive care unit in Germany, for 2018 and 2019 each, and the calculated differences.

## Data Availability

All relevant data are published within the article. Genome sequences of the 31 isolates investigated were available at NCBI GenBank under project number PRJNA721097.

## Abbreviations

ATC: Anatomical Therapeutic Chemical
DDD: Defined daily dose
EUCAST: European Committee on Antimicrobial Susceptibility Testing
HCW: Health-care worker
ICU: Intensive care unit
IQR: Interquartile range
LRSE: Linezolid-resistant *Staphylococcus epidermidis*
MALDI-TOF MS: Matrix-assisted laser desorption/ionization time of flight mass spectrometry
MIC: Minimal inhibitory concentration
MLST: Multilocus sequence typing
ORION: Outbreak reports and intervention studies of nosocomial infections
PD: Patient-days
ST: Sequence type
WGS: Whole-genome sequencing
