# Supplementary material for: Combined antibiotic stewardship and infection control measures to contain the spread of linezolid-resistant Staphylococcus epidermidis in an intensive care unit
Source: Antimicrob Resist Infect Control. 2021 Jun 30;10:99. doi: 10.1186/s13756-021-00970-3 (PMC8242281; doi:10.1186/s13756-021-00970-3)
Supplement: Supplementary file 1 — Additional file 1: Figure S1. Pocket card on the use of antibiotics with Gram-positive coverage, provided during the antimicrobial stewardship intervention on an intensive care unit in southwest Germany, 2018–2020. [file 13756_2021_970_MOESM1_ESM.pptx]

## Slide 1
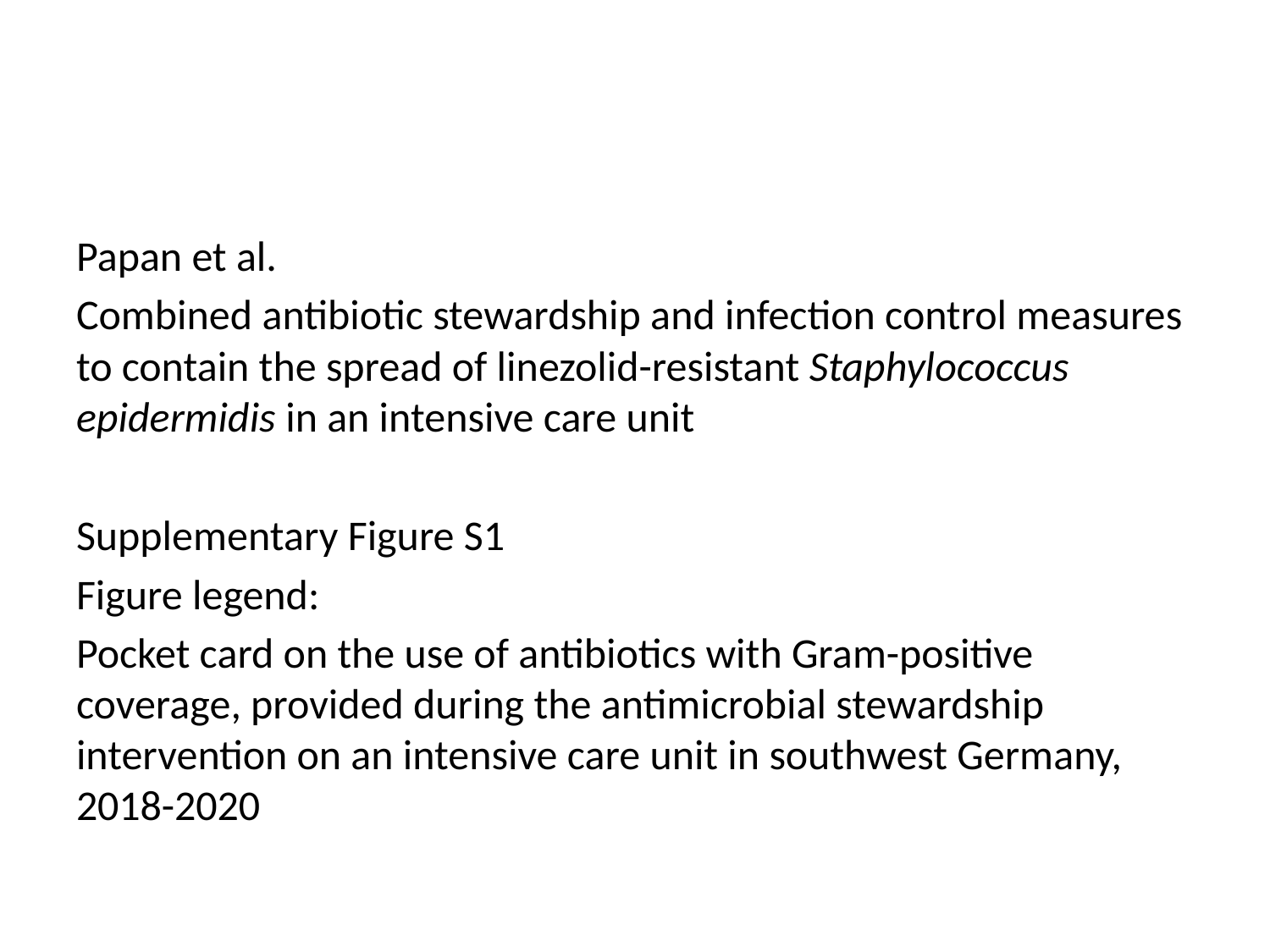

Papan et al.
Combined antibiotic stewardship and infection control measures to contain the spread of linezolid-resistant Staphylococcus epidermidis in an intensive care unit
Supplementary Figure S1
Figure legend:
Pocket card on the use of antibiotics with Gram-positive coverage, provided during the antimicrobial stewardship intervention on an intensive care unit in southwest Germany, 2018-2020

## Slide 2
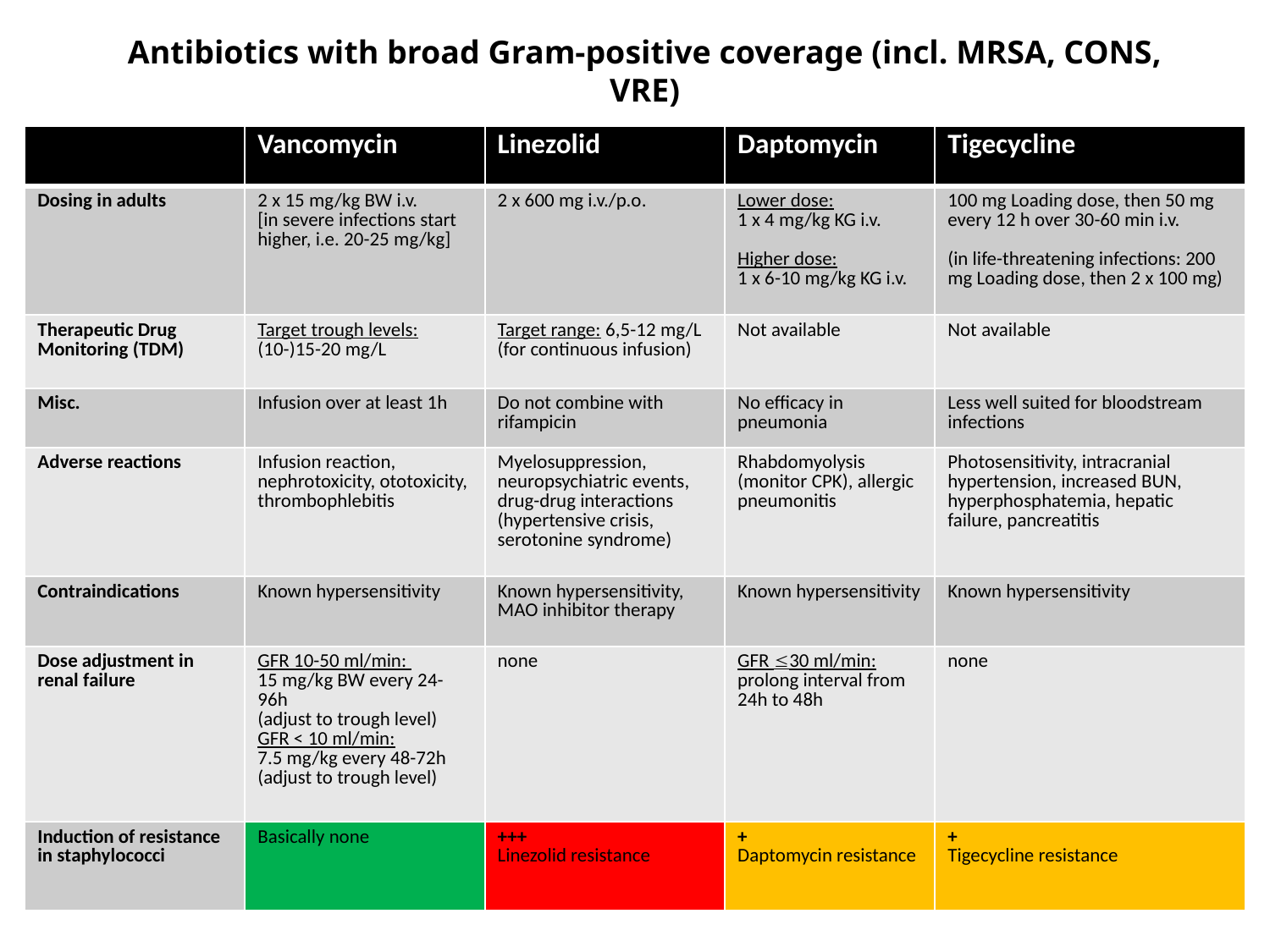

Antibiotics with broad Gram-positive coverage (incl. MRSA, CONS, VRE)
| | Vancomycin | Linezolid | Daptomycin | Tigecycline |
| --- | --- | --- | --- | --- |
| Dosing in adults | 2 x 15 mg/kg BW i.v. [in severe infections start higher, i.e. 20-25 mg/kg] | 2 x 600 mg i.v./p.o. | Lower dose: 1 x 4 mg/kg KG i.v. Higher dose: 1 x 6-10 mg/kg KG i.v. | 100 mg Loading dose, then 50 mg every 12 h over 30-60 min i.v. (in life-threatening infections: 200 mg Loading dose, then 2 x 100 mg) |
| Therapeutic Drug Monitoring (TDM) | Target trough levels: (10-)15-20 mg/L | Target range: 6,5-12 mg/L (for continuous infusion) | Not available | Not available |
| Misc. | Infusion over at least 1h | Do not combine with rifampicin | No efficacy in pneumonia | Less well suited for bloodstream infections |
| Adverse reactions | Infusion reaction, nephrotoxicity, ototoxicity, thrombophlebitis | Myelosuppression, neuropsychiatric events, drug-drug interactions (hypertensive crisis, serotonine syndrome) | Rhabdomyolysis (monitor CPK), allergic pneumonitis | Photosensitivity, intracranial hypertension, increased BUN, hyperphosphatemia, hepatic failure, pancreatitis |
| Contraindications | Known hypersensitivity | Known hypersensitivity, MAO inhibitor therapy | Known hypersensitivity | Known hypersensitivity |
| Dose adjustment in renal failure | GFR 10-50 ml/min: 15 mg/kg BW every 24-96h (adjust to trough level) GFR < 10 ml/min: 7.5 mg/kg every 48-72h (adjust to trough level) | none | GFR ≤30 ml/min: prolong interval from 24h to 48h | none |
| Induction of resistance in staphylococci | Basically none | +++ Linezolid resistance | + Daptomycin resistance | + Tigecycline resistance |
