# Supplementary material for: Combined antibiotic stewardship and infection control measures to contain the spread of linezolid-resistant Staphylococcus epidermidis in an intensive care unit
Source: Antimicrob Resist Infect Control. 2021 Jun 30;10:99. doi: 10.1186/s13756-021-00970-3 (PMC8242281; doi:10.1186/s13756-021-00970-3)
Supplement: Supplementary file 3 — Additional file 3: Table S2. Expenses for the three antibiotics studied during the investigation of an outbreak by linezolid-resistant Staphylococcus epidermidis on an intensive care unit in Germany, for 2018 and 2019 each, and the calculated differences. [file 13756_2021_970_MOESM3_ESM.docx]

**Supplementary Material**

Papan et al.

Combined antibiotic stewardship and infection control measures to contain an outbreak of linezolid-resistant *Staphylococcus epidermidis* in an intensive care unit

Supplementary Table S2

Supplementary Table 2. Expenses for the three antibiotics studied during the investigation of an outbreak by linezolid-resistant Staphylococcus epidermidis on an intensive care unit in Germany, for 2018 and 2019 each, and the calculated differences.

| Substance | expenses 2018 | expenses 2019 | difference (absolute) | difference (relative) |
| --- | --- | --- | --- | --- |
| Daptomycin | 50,273.61 € | 35,426.99 € | -14,846.62 € | -29.53% |
| Linezolid | 5,093.66 € | 2,836.90 € | -2,256.76 € | -44.3% |
| Vancomycin | 252.73 € | 1,073.06 € | +820.33 € | +324.59% |
| Sum | 55,620 € | 39,336.95 € | **-16,283.05 €** | -29.28% |
